# Porcine-Origin Gentamicin-Resistant *Enterococcus* *faecalis* in Humans, Denmark

**DOI:** 10.3201/eid1604.090500

**Published:** 2010-04

**Authors:** Jesper Larsen, Henrik C. Schønheyder, Camilla H. Lester, Stefan S. Olsen, Lone J. Porsbo, Lourdes Garcia-Migura, Lars B. Jensen, Magne Bisgaard, Anette M. Hammerum

**Affiliations:** University of Copenhagen, Frederiksberg, Denmark (J. Larsen, M. Bisgaard); Aarhus University Hospital, Aalborg, Denmark (H.C. Schønheyder); Statens Serum Institut, Copenhagen, Denmark (C.H. Lester, S.S. Olsen, A.M. Hammerum); Technical University of Denmark, Søborg, Denmark (L.J. Porsbo); Technical University of Denmark, Copenhagen (L. Garcia-Migura, L.B. Jensen).

**Keywords:** Keywords: Enterococcus faecalis, high-level gentamicin resistance, pigs, infective endocarditis, molecular typing, MLST, PFGE, zoonoses, bacteria, dispatch

## Abstract

During 2001–2002, high-level gentamicin-resistant (HLGR) *Enterococcus* *faecalis* isolates were detected in 2 patients in Denmark who had infective endocarditis and in pigs and pork. Our results demonstrate that these isolates belong to the same clonal group, which suggests that pigs are a source of HLGR *E*. *faecalis* infection in humans.

Infective endocarditis is a life-threatening infection that involves the endocardial surface or vascular structures in proximity to the heart. Its intrinsic resistance to a number of antimicrobial drugs makes enterococcal infective endocarditis cumbersome to treat. For decades, the mainstay has been the combination of a cell wall–active agent (ampicillin, penicillin, or vancomycin) and gentamicin (*1*). However, high-level resistance to gentamicin hinders the bactericidal activity of combination therapy and increases the likelihood of clinical and microbiologic failure and even death (*2*).

High-level gentamicin-resistant (HLGR) *Enterococcus* *faecalis* has been associated with the hospital setting and prior healthcare exposure, which suggests the existence of a healthcare reservoir (*3*). Nevertheless, enterococci are gut commensals in humans and warm-blooded animals; therefore, reservoirs of HLGR *E. faecalis* conceivably might exist in the community not directly linked to the healthcare setting.

During 2000–2002, the proportion of HLGR *E*. *faecalis* isolates increased from 2% to 6% in the pig population in Denmark (*4**–**6*), which coincided with the emergence of HLGR *E*. *faecalis* isolates among patients with infective endocarditis in North Denmark Region. We undertook this study to determine whether pigs are a source of *E*. *faecalis* infections in humans.

## The Study

Two HLGR *E*. *faecalis* isolates were collected by blood culture (BacT/Alert; bioMérieux, Marcy l’Etoile, France) from separate patients with infective endocarditis in North Denmark Region during 2001–2002 (Table). The regional clinical microbiology laboratory at Aalborg Hospital performed all original antimicrobial susceptibility testing by using Etest (bioMérieux) according to the manufacturer’s recommendations. We retrieved patient information recorded in a regional research database and used it to classify cases as definite, possible, or rejected according to the modified Duke criteria (*8*). A nosocomial origin of infection was determined in accordance with the US Centers for Disease Control and Prevention (*9*).

**Table:** Origins of gentamicin-resistant *Enterococcus* *faecalis* isolates and MICs for gentamicin, Denmark, 2001–2002*

Identification no.	Other name	Origin	Sampling date	MIC for gentamicin, μg/mL	MLST type
124832		IE patient A	2001 Nov 18	>2,048	16
48250		IE patient B	2002 May 2	>2,048	16
7330318-4	D6	Pig	2001 Mar 29	>2,048	**16**
7330612-2	D29	Pig	2001 Jul 25	2,048	**16**
7330948-5	D34	Pig	2001 Nov 23	2,048	**16**
7330115-2		Pig	2001 Jan 29	>2,048	16
7330445-1		Pig	2001 May 21	2,048	16
7430040-2		Pig	2002 Feb 18	1,024	16
7430049-2		Pig	2002 Feb 22	1,024	16
7430275-3		Pig	2002 May 16	>2,048	16
7430291-2		Pig	2002 May 21	2,048	16
7430297-2		Pig	2002 May 22	>2,048	16
7430315-3		Pig	2002 May 23	2,048	35
7430317-5		Pig	2002 May 23	2,048	16
7430328-3		Pig	2002 May 27	>2,048	16
7430405-1		Pig	2002 Jun 24	>2,048	16
7430411-3		Pig	2002 Jun 25	1,024	16
7430416-2		Pig	2002 Jun 24	1,024	16
7430416-3		Pig	2002 Jun 24	>2,048	16
7430803-2		Pig	2002 Nov 18	>2,048	16
7430821-4		Pig	2002 Nov 21	>2,048	16
19116		Pork	2002 Oct 6	>2,048	16
1448		CD human	2003 Oct 1	>2,048	16
3382		CD human	2005 Dec 9	>2,048	16

*MLST, multilocus sequence typing; IE, infective endocarditis; CD, community dwelling. MLST types in **boldface** have been published (*7*).

Patient A, an 81-year-old man with a previous stroke and a urinary catheter, was admitted twice within 1 month with HLGR *E. faecalis* bacteremia, a systolic murmur, transitory cerebral ischemia, and petecchial elements. Aortic valve endocarditis remained a clinical diagnosis because no echocardiography was performed (possible infective endocarditis). The patient died from a new stroke 1.5 months later.

Patient B, a 70-year-old woman with non–insulin-dependent diabetes mellitus, had recurrent bacteremia with HLGR *E. faecalis* and a diagnosis of aortic valve endocarditis confirmed by transesophageal echocardiography (definite infective endocarditis). Aortic valve replacement was performed, and she died 1.5 years later from an unrelated cause. Both cases of infective endocarditis were deemed to be community acquired.

During 2001–2002, a total of 20 HLGR isolates were collected from 19 pigs and from 1 sample of pork as part of the Danish Integrated Antimicrobial Resistance Monitoring and Research Program (DANMAP) (Table) (*5**,**6*). DANMAP also collected fecal samples from humans in the community during 2002–2006. The protocol was approved by the regional Scientific Ethics Committee before the investigation ([KF] 01-006/02). Two HLGR isolates were recovered from community-dwelling persons (Table): a 24-year-old woman (community healthcare worker) and a 2-year-old boy in whom inflammatory bowel disease had been diagnosed and who had received antiinflammatory therapy (mesalazine) within the preceding 30 days. Both regularly ate meat, including pork, and had no contact with food-producing animals during the 7 days before sampling.

MICs were determined for gentamicin (128–2,048 μg/mL) by using the Sensititre system (Trek Diagnostic Systems, East Grinstead, UK) according to current guidelines for inoculation and incubation recommended by the Clinical and Laboratory Standards Institute (*10*). MICs were consequently >1,024 μg/mL (Table).

Multilocus sequence typing (MLST) was performed for the 24 HLGR isolates as described (*11*). The 2 isolates from patients A and B, 18 of 19 isolates from pigs, the isolate from pork, and the 2 isolates from community-dwelling persons belonged to MLST type ST16 (Table). The remaining isolate from pigs belonged to MLST type ST35 (Table).

The major MLST type ST16 was further characterized by pulsed-field gel electrophoresis (PFGE) by using *Sma*I (*12*). The PFGE patterns were analyzed by using BioNumerics version 5.1 (Applied Maths, Kortrijk, Belgium), and isolates were grouped into PFGE clonal types by using Dice coefficients and a value of >82% relatedness (*13*). The PFGE patterns had a minimum of 86% relatedness and were clustered into 1 major clonal group.

The HLGR ST16 isolates were further screened by PCR for the *aac(6′)Ie-aph(2′′)Ia* gene, the *aph(2′′)Ib* and *aph(2′′)Id* genes and the *aph(2′′)Ic* gene to assess the genetic background of gentamicin resistance. All 24 HLGR ST16 isolates carried the *aac(6′)Ie-aph(2′′)Ia* gene and none of the other genes encoding gentamicin resistance.

## Conclusions

Our study provides evidence of the existence of a widespread community reservoir of HLGR ST16 in pigs in Denmark during 2001–2002, which coincided with emergence of HLGR ST16 isolates among IE patients in North Denmark Region. One isolate was present in pork, which supports foodborne transmission, although direct transmission from animals to humans is also possible.

Our study has potential limitations. First, the method used by DANMAP (susceptibility testing of 1 colony per sample, rather than resistance prevalence per sample) may underestimate the extent of the HLGR reservoir in food-producing animals, meat products, and community-dwelling persons. Second, HLGR isolates from patients with infective endocarditis emanated from 2001 and 2002 and therefore do not represent recent trends.

Our findings support the results of a recent study in the United States that identified HLGR *E*. *faecalis* isolates with similar PFGE patterns (<3-band difference) from pork and fecal swabs of outpatients (*14*). These pig-related HLGR isolates, as well as our collection of HLGR ST16 isolates, carry the *aac(6′)Ie-aph(2′′)Ia* gene encoding gentamicin resistance.

Pig-related *E*. *faecalis* isolates belonging to ST16 carry pathogenicity island genes (*7*). These genes are more frequently detected among *E*. *faecalis* isolates recovered from blood or from fecal swabs of inpatients than among isolates from fecal swabs of healthy persons, which suggests that they are associated with invasiveness and virulence in humans (*15*).

With an annual production of >22 million slaughter pigs (*4*–*6*), Denmark has a large potential reservoir of HLGR ST16. Although HLGR ST16 was not detected in other food-producing animals and meat products, this type may not be exclusive to pigs. We found HLGR ST16 isolates in 2 community-dwelling persons during 2003–2005. Preference for eating pork, close contact with the healthcare setting, underlying disease, or a combination thereof may have predisposed these persons to become colonized by this potential pathogen.

HLGR ST16 appears to be transmitted from pigs to humans, although other routes of transmission also may exist. Further studies are needed to better understand the human and veterinary epidemiology of this zoonosis. Areas of study should include recent trends of HLGR among invasive *E*. *faecalis*; size of the reservoir in pigs; its association with antimicrobial drug use in pigs; and whether other animals, immunocompromised persons, or healthy persons constitute a community reservoir of HLGR ST16.
